# Methadone treatments in a Swiss Region, 2001–2008: a registry-based analysis

**DOI:** 10.1186/1471-244X-12-238

**Published:** 2012-12-28

**Authors:** Thérèse Huissoud, Valentin Rousson, Françoise Dubois-Arber

**Affiliations:** 1 Institute of Social and Preventive Medicine, University Hospital Centre and University of Lausanne, Biopole 2 Route de la Corniche 10, CH – 1010, Lausanne, Switzerland

**Keywords:** Opioid substitution, Methadone maintenance, Methadone registry, Treatment duration, Treatment interruption, Switzerland

## Abstract

**Background:**

To determine, in a region of Switzerland, the duration of retention in opioid substitution treatments with methadone (OSTM), duration of treatment interruptions, probability of re-entry to treatment after a treatment interruption, and associated factors.

**Methods:**

A secondary analysis of registry-based data was performed with patients (*n* = 2880) registered in the methadone treatment register database of the Public Health Service of the canton of Vaud between January 1, 2001 and June 30, 2008. Survival analysis and multivariate analysis was conducted.

**Results:**

The probability of remaining on treatment was 69% at 1 year and 45% at 3 years (*n* =1666). One-third of patients remained on treatment beyond 5 years. The estimated hazard of leaving treatment was increased by a ratio of 1.31 in the case of a first treatment (*P* = 0.001), 1.83 for those without a fixed home (*P* < 0.001), and 1.29 for those younger than 30 years old (*P* < 0.001). The probability of having begun a new treatment after a first interruption was 21% at one year, 38% at 3 years, and 43% at 5 years (*n* = 1581). Factors at the interruption of treatment associated with a higher probability of re-entering were: interruption not due to methadone withdrawal, bad physical health, and higher methadone dose.

**Conclusions:**

OSTM are long-term (maintenance) treatments in Switzerland. Younger age, bad living conditions at entry, and first treatment are predictors of lower retention. Approximately one-half of patients who interrupt treatment will re-enter treatment within 5 years.

## Background

Opioid substitution treatments with methadone (OSTM) have markedly developed in Switzerland (about 7.66 million inhabitants) increasing from approximately 5000 patients on treatment in 1989 to more than 18 000 in 2000 [1]. In 2008, more than 16 500 persons were on OSTM in Switzerland [2], among an estimated number of opiate-dependent persons in Switzerland of 25 000 [3]. The decrease between 2000 and 2008 is estimated to be due to a decrease in the number of new heroin consumers. This evolution has also occurred in other European countries; for example, in Norway, the number of treatments rose from less than 500 in 1998 to 3000 in 2004 [4].

First conceived as a therapeutic response that was limited in time and ended with a withdrawal from methadone [5], OSTM progressively evolved towards maintenance treatments that are considered necessary over the long-term (methadone maintenance treatments, MMT) [6]. The current representation of drug dependence is that of a chronic disease with repeated phases of consumption, abstinence, and treatment [7]. Periods of abstinence are mostly short [8,9] and are often followed by relapse [10]. In Switzerland, a therapeutic approach based on patients’ needs is currently recommended [11], and most OSTM are probably MMT.

In the Swiss canton of Vaud (about 730 000 inhabitants), the number of OSTM followed the same trend of increase and stabilisation: since 2000, approximately 1600 patients have benefitted from an OSTM. Treatments require an authorisation from the chief cantonal medical officer, and are administered mainly by general practitioners (GPs) in private practice who are allowed to begin and regularly follow MMT. The two specialized drug treatment centres follow only one-third of all patients. Access to treatment is unlimited and treatments are mainly oriented towards long-term MMT. Treatments have to be in accordance with the guidelines [12] laid down by the Cantonal Health Department regarding treatment induction, doses, mode of delivery, controls, security, rules for “take home” formulations, etc. Documents of entry to treatment or prolongation of treatment have to be filled in by the GP requiring a prescription authorization (see below under methods). Methadone in 1% solution (syrup) is the substance of reference, but buprenorphin or – rarely - oral slow release morphine may also be prescribed, according to the guidelines from the Swiss Society of Addiction Medicine. These last two products are very seldom used. Medical doctors requiring a treatment authorization from the chief cantonal medical officer for the first time have to engage in the continuous educational programme on methadone. Evaluation of abstinence from drug consumption is regularly conducted (e.g. with urine controls). Continued drug use has to be reported to the authority but is not a compulsory reason for removing the patient from treatment.

Many studies have analysed treatment retention in OSTM [4,13-20] or factors associated with retention [21,22]. Waal’s evaluation of substitution treatments in Norway showed a high retention rate in spite of very restrictive treatment rules, with less convincing results regarding treatment outcome (rehabilitation) [4]. According to Magura et al. [22], factors associated with retention are mainly related to treatment characteristics: only two of 16 pre-treatment variables, compared with five of six during-treatment variables, had significant effects on retention. Others have analysed the periodicity of treatment(s) and relapse(s) over time: as reviewed by Magura et al. in 2001 [23] and Amato et al. in 2005 [24], a high rate of relapse after the end of methadone treatment and a rapid return to treatment have been described. In a cohort analysis in New South Wales, Burns et al. reported an estimate of 197 days for treatment duration and a mean of 2.5 treatment episodes per patient [14].

Few studies have analysed the patterns of treatment history - i.e. the occurrence of several consecutive treatment episodes with interruptions in-between - or the duration of treatment interruptions. In Alicante [25], a survival analysis conducted on patients on MMT demonstrated that 16% of patients had more than one treatment episode, with a median interruption duration of 13 months. Patients who re-enrolled had longer total time within MMT than patients retained in treatment and drop-outs. This is also apparent in the study of Nosyk et al. [26].

The objectives of this study are – by using registry-based data:

● To estimate the duration of retention in MMT (median duration and probability of staying in treatment at different times), and the factors present at the initiation of the treatment that are associated with treatment duration.

● To estimate the duration of interruption, the probability of re-entry to treatment after a first interruption of treatment, and the factors present at the end of the first treatment period that are associated with re-entry.

## Methods

In the canton of Vaud, methadone treatment can be prescribed and prolongations obtained only with a compulsory official authorization from the Chief cantonal medical officer, and each authorization (“entry”) “prolongation” or “end of treatment” document is retained in the methadone treatment register of the canton of Vaud. The analysis includes completely anonymized data of all patients registered in the methadone treatment register database of the Public Health Service of the canton of Vaud between January 1, 2001 and June 30, 2008. As a result, no connection to any other database such as the death register is possible. The database only contains a patient number for each patient; the corresponding name is retained in the Public Health Service of the canton of Vaud and remained unknown to the researchers. This research received the approval of the Chief medical officer of the canton. It is a secondary and retrospective analysis of anonymous data and obtaining patient consent in this situation is not possible.

Each patient can have three types of documents recorded in the register under his/her patient number:

● “entry” documents, which are completed by the doctor at each new entry to treatment, to receive authorization from the Chief medical officer;

● “requests for a prolongation of treatment authorization”, which are completed by the doctor every 12 months; several requests for prolongation may follow, year after year;

● “end of treatment” documents, which are completed by the doctor in cases of treatment interruption and include the reasons for interruption: true end of treatment with methadone withdrawal or interruption of treatment for other reasons, or transfer to another doctor.

Each doctor following a patient on methadone must complete these documents; data are sent to the Public Health Service which records them in the registry and then delivers the treatment authorization.

In case of transfer to another doctor, a relatively long period may elapse before the new doctor sends the new “entry” document, even if the patient continues MMT. Because of this administrative delay up to 2 months before the registration of the new treatment document, we have considered in this analysis a period of more than two months to define a treatment interruption.

The following variables were included in the analysis: age, gender, living conditions (fixed abode, without fixed abode, or institution including prison), source of income (full-time or part-time employment, social insurance, social aid), had a previous methadone treatment (yes/no), currently injecting (yes/no), health (HIV test [yes/no-not known] and HIV status [positive/negative-unknown], HCV test [yes/no-not known] and HCV status [positive/negative-unknown], physical condition [good/bad], psychological condition [good/bad] and social situation [good/bad], as estimated by the doctor without standardized rating system), treatment administration mode (only 2 mentions proposed without more precision: methadone delivered each day or 1 to 4 times each week), and methadone dose (maintenance dose). We chose not to include the available variable “drugs use during the treatment (heroin, cocaine/daily, occasionally, no use during last month)” because of the bad quality of the data (many missing data). The “end of treatment” document includes in addition reasons for stopping: dropping out, methadone withdrawal, move to another canton, transfer to another doctor, entry into prison, death, other. It also includes the daily dose of methadone at the time of interruption.

### Statistical analysis

The duration of treatment and the duration of interruption were estimated using the Kaplan-Meier method. Their statistical association with various factors of interest was assessed using a log-rank test. *P*-values smaller than 0.05 were considered statistically significant.

The data were processed using SPSS software (version 15.0) for Windows (SPSS Inc., Chicago, Illinois 60606).

**Figure 1:**
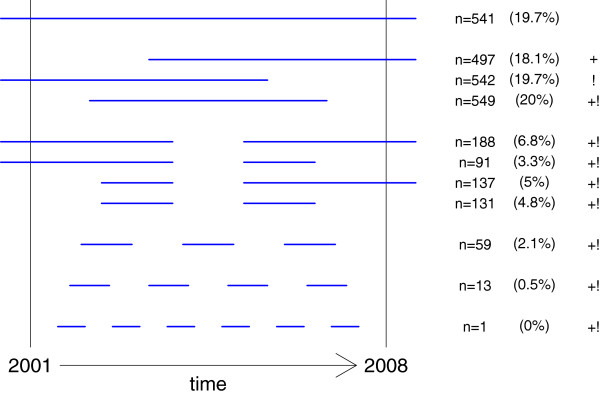
**Number of patients by pattern of treatment history, 2001–2008.** Each line represents a pattern of treatment history with the number and proportion of patients exhibiting this pattern. The length of the line is not proportional to the duration of treatment, only the fact that the line is cutting or not the lower or the upper limit of the observation (January 1^st^ 2001, June 30, 2008) is of interest. For example, - the first line represents the pattern “having had one treatment episode, beginning before January 1^st^, 2001, and ending after June 30th 2008”, n =541 (19.7%). - the second line represents the pattern “having had only one treatment episode, beginning between January 1^st,^ 2001, and ending after June 30th, 2008”, n = 497 (18.1%). - the last line represents the pattern “having had more than 4 treatments episodes beginning after January 1^st,^ 2001, ending before June 30th, 2008”, n = 1 (0.0%). + group of individuals included in the analysis of treatment duration. ! group of individuals included in the analysis of treatment interruption.

The survival analysis of being in treatment was calculated from the first entry questionnaire registered after January 1, 2001. Each patient having at least one entry questionnaire between January 1, 2001 and June 30, 2008 was included in the analysis, (i.e., for 1666 patients; *n* = 497 + 549 + 188 + 91 + 137 + 131 + 59 + 13 + 1 = 1666, see Figure 1). For patients who had several treatment episodes (an episode being a period of treatment without interruption), only the first treatment episode was included in the analysis, i.e. the unit of analysis was the patient.

The probability of re-entering treatment after an interruption (i.e. having a new document of entry to treatment) was calculated from the date of the first “end of treatment” document registered for each patient. One thousand seven hundred and eleven patients had an interruption of treatment attested by an “end of treatment” document established between January 1, 2001 and June 30, 2008 (*n* = 542 + 549 + 188 + 91 + 137 + 131 + 59 + 13 + 1 = 1711, see Figure 1 which shows the distribution of the various patterns of treatment history). However, patients for whom the reason for the interruption of treatment was death were excluded from the analysis (*n* = 130) because they had no chance of coming back. Therefore, the probability calculation was conducted on only 1581 patients.

A multivariate analysis to test for the simultaneous association of various factors with the duration of treatment or interruption was carried out using a Cox regression model.

## Results

### Population

Overall, 2880 different patients were registered in the database between January 1, 2000 and June 30, 2008. For 87 patients, the documentation was insufficient to determine the evolution of treatment (only one document was found); these patients were excluded from the analysis. For 44 patients, the dates and sequence of the various documents of “entry”, “prolongation”, and “end of treatment” were chronologically aberrant; these patients were also excluded.

The analysis was conducted with the remaining 2749 patients for whom several documents were available, including one “entry” or one “end of treatment” document. Among these 2749 patients, 541 (19.7%) had begun treatment before January 1, 2001, had had a prolongation document for each year until 2008 and continued their treatment without interruption until June 30, 2008 (Figure 1).

A majority of patients (57.8%) had only one treatment episode spanning the entire period, a treatment episode being a period of treatment without any interruption. Among them, 497 patients had only an “entry” document followed by several “prolongation” documents because their treatment episode was still ongoing at the end of the study period. Similarly, 542 patients had only an “end of treatment” document after several “prolongation” documents because during the study period, they completed a treatment episode begun before 2001 and had not re-entered treatment by June 30, 2008. Five hundred forty-nine persons had a single “entry” document followed or not by “prolongation” documents and an “end of treatment” document recorded during the period: these individuals began and completed a single treatment episode between 2001 and 2008.

Five hundred forty-seven patients (19.9%) had two treatment episodes during the study period; among them, 279 patients initially had an “end of treatment” document, which was followed by the beginning of a new treatment (still ongoing at June 30, 2008 for 188 individuals). Other patients (*n* = 268) began a treatment, stopped it, and entered again (for 137 of these patients, this episode was still ongoing at the end of the study period, while 131 had finished their second treatment episode). A small proportion of patients (2.6%) had more than two treatment episodes: 59 persons had three, 13 persons had four, and one person began and ended treatment six times during the study period.

### Treatment duration

The survival analysis for the first treatment episode reveals that, of the 1666 patients registered as beginning treatment during the study period, 982 finished this treatment episode before June 30, 2008. Reasons for having an “end of treatment” document included interruption (for various reasons) or the actual end of treatment (withdrawal from methadone). The probability of remaining on treatment was 69% at 1 year and 45% at 3 years; one-third of patients remained on treatment beyond 5 years (Table 1).

**Table 1:** Treatment duration and associated characteristics at entry, 2001-2008

	**Total *N***	Number of events*	**Median (days)**	**In treatment at 1 year %**	**In treatment at 3 years %**	**In treatment at 5 years %**	***P*-value**
**All**	1666	982	898	69	45	34	
**Had a previous treatment**							
Yes	1314	765	982	71	50	37	**0.001**
No	352	217	643	63	36	27	
**Sex**							
Male	1190	711	868	69	42	32	0.088
Female	476	271	1039	73	50	38	
**Age**							
≤30 years	700	454	718	64	41	28	**< 0.001**
>30 years	965	527	1037	72	50	40	
**Living conditions**							
Fixed abode	1282	715	1091	74	50	36	**<0.001**
Without fixed abode	160	110	377	50	27	25	
Institution/Prison	189	138	434	52	27	23	
**Full-time job**							
No	1260	741	876	70	48	32	0.925
Yes	406	241	957	72	48	37	
**Social insurance**							
No	1435	858	889	68	43	33	0.093
Yes	231	124	1117	74	51	39	
**Social aid**							
No	1199	711	888	71	43	34	0.661
Yes	467	271	916	71	43	36	
**Good social condition**							
No	1241	741	868	71	43	34	0.263
Yes	425	241	1078	71	51	39	
**Good psychological condition**							
No	1239	741	888	71	43	34	0.26
Yes	427	241	960	71	47	39	
**Good physical condition**							
No	751	456	887	71	43	34	0.726
Yes	915	526	916	71	43	34	
**Hepatitis C status**							
Negative or Unknown	1132	660	909	72	46	32	0.69
Positive	534	322	869	68	44	36	
**HIV status**							
Negative or Unknown	1562	918	909	70	50	32	0.852
Positive	104	64	779	71	42	38	
**Currently injecting**							
No	1065	602	946	72	41	35	0.089
Yes	601	380	792	63	48	35	
**Number of methadone deliveries per week**							
1 to 4	454	236	1061	75	49	37	**0.009**
>4	1060	646	842	67	43	32	
**Daily methadone dosage**							
0.1 to 30 mg	450	260	842	68	40	29	0.407
31 to 50 mg	507	297	1057	72	48	34	
51 to 75 mg	335	191	869	70	46	38	
>75 mg	318	192	918	68	44	34	

* An event is defined by the interruption of treatment, i.e. having an “end of treatment” document.

**Figure 2:**
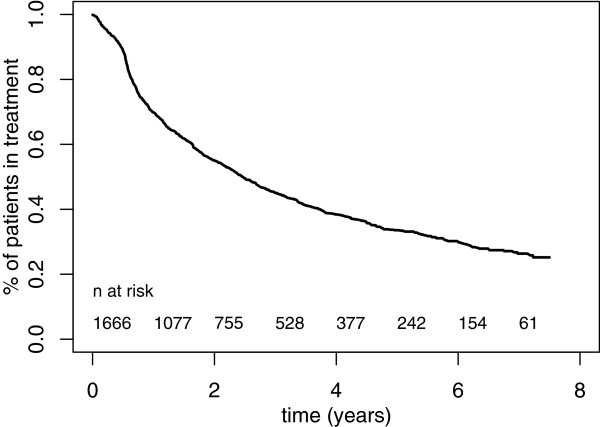
Proportion of patients in treatment, 2001–2008 (n = 1666).

**Figure 3:**
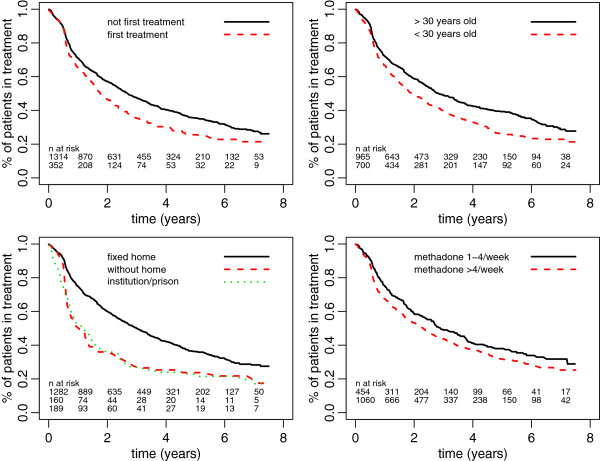
**Proportion of patients in treatment with respect to treatment status, age, living conditions, and methadone delivery mode at entry to treatment, 2001–2008.** N at risk: first line refers to solid line; second line refers to dashed line; third line refers to dotted line.

The duration of treatment varied with regard to a range of factors present at treatment initiation (Table 1 and Figures 2 and 3). Variables associated with shorter treatment duration were: participating in the first treatment episode, living without a fixed abode or living in an institution/prison, being younger than 30 years old, and having methadone delivered 4 times a week. Methadone maintenance dosage reached at the beginning of treatment, state of health as estimated by the doctor, source of income, and gender were not associated with treatment duration.

When the treatment was a first treatment episode, the interruption of treatment (regardless of reason) came earlier: 63% of patients undergoing the first treatment episode remained on treatment at one year (36% at 3 years), while 71% of patients who had been in treatment previously, remained on treatment at one year (50% at 3 years) (*P* = 0.001). Fifty percent of patients aged 30 years and older remained on treatment after 3 years, compared with only 40% of patients younger than 30 years of age. It was estimated that 50% of persons living in a fixed abode at the beginning of treatment would still be on treatment after 3 years, compared with only 27% of people not living in such conditions (*P* < 0.001). For patients living without a fixed abode, the probability of leaving treatment was particularly high. When the doctor reported, at the beginning of treatment, that the patient could receive his/her methadone doses for several days, the probability of being on treatment after 3 years was higher (*P* < 0.001).

In a multivariate Cox regression model including the four factors that were significant in the univariate analyses above, the estimated hazard of ending the treatment was increased by a ratio of 1.31 for participants in a first treatment (*P* = 0.001), by a ratio of 1.82 for those lacking a fixed abode (*P* < 0.001), and by a ratio of 1.29 for patients younger than 30 years old (*P* < 0.001). Having methadone delivered each day was no longer significant (*P* = 0.52). We checked for interaction between the mode of delivery and living condition: there was no significant interaction between these two factors (*P* = 0.13).

### Probability of re-entering treatment after an interruption

**Figure 4:**
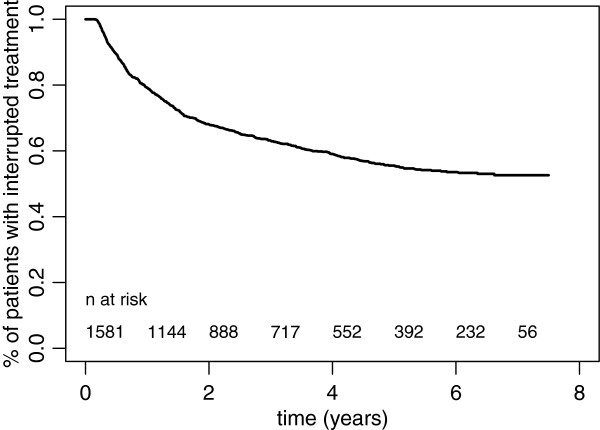
Duration of intervals between treatment episodes, 2001–2008 (n = 1581).

**Figure 5:**
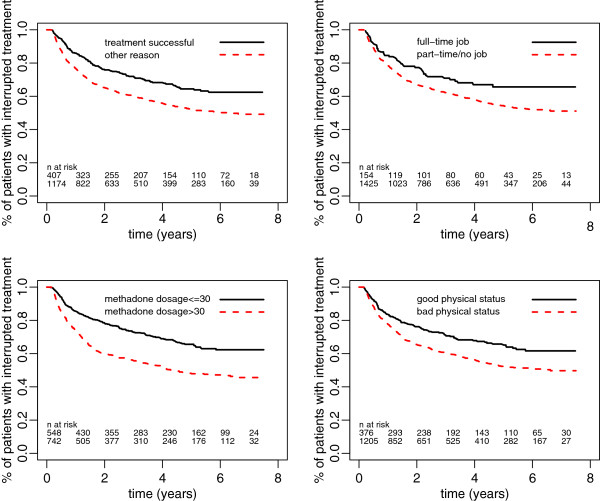
**Duration of intervals between treatment episodes with respect to reason for ending treatment, occupation, methadone dosage, and physical status at treatment interruption, 2001–2008.** N at risk: first line refers to solid line; second line refers to dashed line.

The probability of having begun a new treatment episode, calculated for 1581 studied patients, was 21% at one year, 38% at 3 years, and 43% at 5 years. After 5 years, the probability of re-entry became very small, with a flattening of the survival curve of interruption (Table 2 and Figures 4 and 5). The probability of having begun a new treatment episode differed according to the reasons for interrupting treatment and the characteristics of the patient at the time of the interruption. Among patients who had finished their treatment and been withdrawn from methadone, the probability of re-entering treatment after one year was 15%; among patients considered drop-outs at the moment of interruption, this probability rose to 25% (*P* < 0.001). After 3 years, it is estimated that 42% of drop-outs are back in treatment, compared with 29% of persons ending treatment with a withdrawal from methadone. A higher maintenance methadone dosage at the time of interruption was significantly associated with the probability of returning to treatment (*P* < 0.001).

**Table 2:** Duration of intervals between treatment episodes and associated characteristics at treatment interruption, 2001–2008

	**Total *N***	Number of events*	**Percentile 75 (days)**	**Out of treatment at 1 year %**	**Out of treatment at 3 years %**	**Out of treatment at 5 years %**	***P*-value**
**All**	1581	618	478	79	62	57	
**Sex**							
Male	1118	445	455	79	62	56	0.395
Female	461	173	505	80	64	59	
**Age**							
≤30 years	591	235	432	79	63	56	0.886
>30 years	986	382	491	79	63	58	
**Living conditions**							
Fixed abode	482	178	612	82	65	58	0.996
Without fixed abode	30	12	501	87	62	57	
Institution/Prison	149	53	496	78	65	60	
**Full-time job**							
No	1425	572	448	77	61	56	**0.011**
Yes	154	46	806	82	72	66	
**Social insurance**							
No	1460	582	466	77	62	57	0.071
Yes	119	36	547	71	72	65	
**Social aid**							
No	1377	533	474	79	63	57	0.674
Yes	202	85	496	79	59	67	
**Good social condition**							
No	1388	567	442	77	60	56	**<0.001**
Yes	193	51	1215	89	77	71	
**Good psychological condition**							
No	1350	547	442	77	60	54	**0.003**
Yes	231	71	960	84	75	64	
**Good physical condition**							
No	1205	500	433	77	60	53	**<0.001**
Yes	376	118	794	83	71	64	
**Hepatitis C status**							
Negative or Unknown	1316	510	488	79	63	57	0.753
Positive	263	108	445	72	60	57	
**HIV Status**							
Negative or Unknown	1530	597	484	79	62	57	0.519
Positive	49	21	395	72	60	57	
**Currently injecting**							
No	1470	572	480	79	63	57	0.767
Yes	111	46	433	79	61	57	
**Reason for stopping treatment**							
Drop-out	488	217	391	75	58	48	**<0.001**
Methadone withdrawal	407	125	826	85	71	63	
Other reason	686	276	410	77	61	55	
**Daily methadone dosage**							
0.1 to 30 mg	548	165	945	85	73	65	**<0.001**
31 to 50 mg	272	124	390	77	58	48	
51 to 75 mg	204	101	289	72	53	45	
>75 mg	266	120	312	72	55	49	

* An event is defined by re-entry to treatment, i.e.having a new document of entry to treatment.

Having a full-time job at the time of treatment interruption (*P* = 0.011), a good state of psychological health (*P* = 0.003) or physical health (*P* < 0.001), and a good social situation (*P* < 0.001) were significantly associated with a higher probability of not returning to treatment.

In a multivariate Cox regression model including the six factors that were significant in the univariate analyses above, the fact of having a full-time job was no longer significant (*P* = 0.97), as were social condition (*P* = 0.22), xand psychiatric condition, *P* = 0.42), while having a bad physical condition was just significant (*P* = 0.011). When applying a backward selection method, we could find a model including three significant factors; the estimated hazard of re-entering treatment was increased by a factor of 1.32 if treatment had not been successful (*P* = 0.02), by a ratio of 1.48 for patients in a bad physical condition (*P* < 0.001), and by a factor of 1.59 if the methadone dosage was more than 30 mg per day (*P* < 0.001).

## Discussion

This study confirms that OSTM are actually long-term treatments in the studied region of Switzerland. A majority of patients followed their treatment continuously: among the patients registered between 2001 and 2008, 77.5% had only one treatment episode during the study period, including a group (19.7% of all patients) that had initiated treatment before the beginning of the study period (i.e., before January 1, 2000) without any interruption until June 30, 2008, the date of the end of the study period. Among those who started a treatment episode between January 1, 2000 and the end of the period, 69% remained in treatment after one year and 45% after two years. The risk of interrupting treatment appeared higher during the first 12 months of treatment.

The duration of treatment retention varies widely across different studies. A German study [18] observed that 50% of patients continued their treatment for more than 7 years, with a 65% retention rate at one year. Another study in Spain reported similar results, with a 60% retention rate at one year and 38% at 3 years [25]. In a recent US study, shorter treatment duration (mean 8 months) and lower retention rates were observed [13]. Low retention rates at one year (25–50% in certain studies) were reported in a 2001 review by Magura et al. [23], who identified the restrictive and coercive nature of specific treatment regimens as factors associated with low retention rates. A study of the treatment system in Norway [4] that demonstrated low retention rates resulted in the loosening of conditions of access to treatment and a modification of exclusion rules. A recent Italian longitudinal study conducted on more than 5000 patients [27] concluded that abstinence-oriented treatments were associated with lower retention rates than substitution-oriented treatments. A previous study had also demonstrated poorer outcomes with methadone reduction regimens than with methadone maintenance regimens [20].

The high retention rate measured in the canton of Vaud may be linked with the availability of a wide range of substitution treatment options, from treatment with abstinence objectives (high-threshold treatments) to treatment with risk reduction objectives (low-threshold treatments). This high retention rate is expected to improve patients outcomes in particular in terms of decreasing heroin consumption, decreasing i/v drug use and associated risks [28].

In our study, the probability of interrupting treatment was higher in cases of first treatment episode, in persons without a fixed abode, and in persons younger than 30 years of age. Bell et al. already identified lower retention rates in cases of first treatment episode [15], and lower age has been associated with lower retention rates in several studies [13,16,22]. Health-related variables and methadone dose were not associated with treatment duration, although methadone dose has been identified as a factor predictive of retention [16,29]. It is likely that the methadone dosage reported in the entry document in our study does not reflect the final maintenance dosage. In another study using registry data, conducted in Ontario, Canada, where methadone treatment may also be administered by general practitioners, the probability to have longer treatments episodes (730 days or more) was negatively associated with the number of episodes [30].

The duration of the interruption between two treatment episodes has not been studied, although it is known that many patients experience several treatment episodes [25,31-33]. We observed that, after a treatment interruption, a one-year probability of re-entering treatment of 21% and a five-year probability of 43%; the probabilities were lower when the treatment interruption was a “true” end of treatment featuring methadone withdrawal. After five years, the probability of re-entering was very low regardless of the reason for interruption.

Gossop et al. [32] observed that 50% to 60% of persons who had stopped treatment had re-entered into one or another modality of treatment. Ball and Ross [33] estimated that approximately 70% of patients who ended a treatment with a withdrawal from methadone had either re-entered treatment or resumed consumption of opiate drugs of abuse.

Three factors at the interruption of treatment were associated with a higher probability of re-entering: an interruption not due to methadone withdrawal, bad physical health, and higher methadone dose. Bell et al. [34] demonstrated that the significant predictors of re-entry to treatment were younger age and shorter duration of the first treatment episode.

The long treatment durations associated with high rates of re-entry after interruption observed in our study contribute additional thoughts to the debate regarding the real possibilities of methadone withdrawal in OSTM. In light of the known protective effects of methadone maintenance on mortality [35,36], some professionals question the relevance of methadone withdrawal, particularly whether it would be an inappropriate solution for many patients on MMT [5]. In this context, Clausen et al. [36] insist on the importance of retaining patients in treatment or rapidly reintegrating them in cases of interruption. Strike et al. [30], in their study of predictors of retention, suggest that repeat episodes may not be as beneficial as previous research reports.

In our study, the probability of re-entering was associated with bad conditions at the time of interruption and most cases who re-entered did so within one year, suggesting that drop-outs have the capacity to reintegrate rapidly and therefore to reduce their risks (in particular linked with injection). However, a non-negligible proportion of patients who interrupted treatment were still out of treatment after five years, and even if we do not know anything about their situation (particularly regarding mortality after interruption for another cause), it is possible that successful methadone withdrawal occurred in a part of this group. However, it was not possible to link this database with other databases, especially on mortality. More research is still needed to assess long-term outcomes after treatment interruption (regardless of cause) in long-term MMT.

Our study has limitations. The first limitation is that the analysis covers only one region of Switzerland. However, this region represents 10% of the population, and treatment recommendations issued by the Federal Office of Public Health are applied in the canton of Vaud.

The probability of staying in treatment was calculated on the basis of the first “entry” document registered during the considered period. Many of these treatments are not first treatments (the information is available) and this overestimates the general probability of staying in treatment since the analysis shows a lower retention for first treatments. We nevertheless chose this option to increase the number of patients in the model.

Because long delays have been observed in the administrative transmission of documents when the patient was transferred to a different doctor [37], we considered that when the duration of the interruption was less than 2 months, there was no treatment interruption. We may have overestimated treatment retention and underestimated the number of drop-outs by doing so. Furthermore, the register only includes patients living in the canton of Vaud. When a person begins treatment, a previous treatment in another canton may not be mentioned. This would lead to a possible underestimation of the duration of treatment.

The poor quality of data on drug use during the treatment was another limitation to the analysis of the database, as was the fact that the assessment of the psychological and physical state of the patient as well as its social inclusion was left to physician appreciation without standardized measurement. The quality of the information on socio-demographic variables and other health information (HIV/ hepatitis) was good. Overall, we feel that these registry-based data are robust and provide useful information to health providers.

## Conclusions

OSTM are long-term (maintenance) treatments in Switzerland. Younger age, bad living conditions at entry, and first treatment are predictors of lower retention. Approximately one-half of patients who interrupt treatment will re-enter treatment within 5 years.

## Abbreviations

OSTM: Opioid substitution treatments with methadone; MMT: Methadone maintenance treatments; GPs: General practitioners.

## Competing interests

The authors declare no competing interests.

## Authors’ contributions

All the authors have contributed substantially. TH participated in the conception of the study and of the analysis, managed literature search, prepared the database, analysed the data, and prepared the first version of the manuscript. VR supervised the analysis of the data, and conducted specific parts of it. FDA participated in the conception of the study and of the analysis, participated in the analysis. All 3 authors discussed and revised the manuscript, approved the final manuscript and the submission of the manuscript.

## Pre-publication history

The pre-publication history for this paper can be accessed here:

http://www.biomedcentral.com/1471-244X/12/238/prepub
